# Identification of a type I nitroreductase gene in non-virulent *Trypanosoma rangeli*

**DOI:** 10.1590/0074-02760160532

**Published:** 2017-07

**Authors:** Marjorie Montenegro, Claudia Cuervo, Constanza Cardenas, Silvia Duarte, Jenny R Díaz, M Carmen Thomas, Manuel C Lopez, Concepcion J Puerta

**Affiliations:** 1 Pontificia Universidad Javeriana, Facultad de Ciencias, Departamento de Microbiología, Laboratorio de Parasitología Molecular, Bogotá, Colombia; 2 Consejo Superior de Investigaciones Científicas, Instituto de Parasitología y Biomedicina López Neyra, Granada, Spain; 3 Pontificia Universidad Católica de Valparaíso, Núcleo de Biotecnología Curauma, Valparaíso, Chile

**Keywords:** type I nitroreductase, Trypanosoma cruzi, Trypanosoma rangeli

## Abstract

Trypanosomatid type I nitroreductases (NTRs), i.e., mitochondrial enzymes that metabolise nitroaromatic pro-drugs, are essential for parasite growth, infection, and survival. Here, a type I NTR of non-virulent protozoan *Trypanosoma rangeli* is described and compared to those of other trypanosomatids. The *NTR* gene was isolated from KP1(+) and KP1(-) strains, and its corresponding transcript and 5’ untranslated region (5’UTR) were determined. Bioinformatics analyses and nitro-drug activation assays were also performed. The results indicated that the type I NTR gene is present in both KP1(-) and KP1(+) strains, with 98% identity. However, the predicted subcellular localisation of the protein differed among the strains (predicted as mitochondrial in the KP1(+) strain). Comparisons of the domains and 3D structures of the NTRs with those of orthologs demonstrated that the nitroreductase domain of *T. rangeli* NTR is conserved across all the strains, including the residues involved in the interaction with the FMN cofactor and in the tertiary structure characteristics of this oxidoreductase protein family. mRNA processing and expression were also observed. In addition, *T. rangeli* was shown to be sensitive to benznidazole and nifurtimox in a concentration-dependent manner. In summary, *T. rangeli* appears to have a newly discovered functional type *I NTR*.

Type I nitroreductases (NTRs) are oxygen-insensitive oxidoreductases that contain flavin mononucleotide (FMN) as a cofactor. They catalyse a series of two-electron reduction reactions using nicotinamide adenine dinucleotide hydrogen (NADH) as the electron donor and nitroaromatic compounds and quinones as substrates (Zenno et al. 1996). They share some characteristic signatures, such as a nitroreductase domain and residues critical for cofactor interactions (R74, S76, R78, Q131, G247, and F248) (Parkinson et al. 2000). Recently discovered in eukaryotes, type I NTRs play an essential role in nitro-drug activation and resistance in several protozoans of clinical significance, including *Trypanosoma cruzi*, *Trypanosoma brucei*, *Leishmania donovani*, *Leishmania major*, *Giardia lamblia*, *Entamoeba* spp., and *Trichomonas* spp. (Wilkinson et al. 2008, Pal et al. 2009, Müller et al. 2013, Voak et al. 2013, Wyllie et al. 2013). In trypanosomatids, NTRs are mitochondrial enzymes that appear to be essential for parasite growth and survival (Wilkinson et al. 2008, Voak et al. 2013, Wyllie et al. 2013) though the precise biological role remains unknown. Nonetheless, findings about its quinone substrate preference, similarity to FMN-dependent NADH dehydrogenases (Hall et al. 2012), and the relationship with nitro-drug action and ubiquinone biosynthesis (Alsford et al. 2012) suggest that this enzyme functions as a mitochondrial ubiquinone reductase to mediate reduction of ubiquinone to ubiquinol via NADH and to maintain the NADH/NAD^+^ balance in this organelle (Wilkinson et al. 2008).

Given the importance of type I NTRs for pathogenic trypanosomatids, the presence of a type I NTR in avirulent parasite *T. rangeli* is described and discussed here, with a comparison to the enzymes from other trypanosomatids. The *T. rangeli NTR* gene was amplified by polymerase chain reaction (PCR) using genomic DNA from the KP1(-) Tre isolate (Puerta et al. 2009) and the NTR-FW (5’-GAG AAA TGG CAT AAA AAG AGG CC-3’) and NTR-RV (5’-AAA ACT TTC CCC ACC GAA CCA-3’) primer pair. Multiple sequence alignment of the pGEM^®^-T Easy vector-cloned sequence (GenBank accession number AHI85557) with that of putative type I NTR*-*coding genes from CL Brener and *T. cruzi* 058PUJ strains (Supplementary data, Table I) revealed identity percentages of 71.6% and 72.4%, respectively. To compare the NTR sequence between different *T. rangeli* groups, the corresponding coding sequence from the KP1(+) Choachí isolate was also PCR amplified, cloned, and sequenced (GenBank accession number AHI85556). Identity of 98.3% was observed between the nucleotide sequences from the KP1(-) Tre and KP1(+) Choachí isolates, and 97.5% identify was observed between Tre and the reported Brazilian SC-58 KP1(-) sequence (Sincero et al. 2015). These results are in agreement with a recent report of the existence of an additional *T. rangeli* group composed of KP1 (-) strains isolated from the Brazilian Amazon Region, including SC58.

To investigate whether *T. rangeli* type I *NTR* is expressed at the mRNA level and undergoes maturation, reverse transcription (RT)-PCR was performed using cytoplasmic mRNA from *T. rangeli* Tre epimastigotes (Montenegro et al. 2013). cDNA was synthesised using the oligo (dT)_15_ primer and NTR-FW- and NTR-RV-specific primers to amplify the NTR-coding sequence or the SL/TcTr (5’-AAC TAA CGC TAT TGA TAC AGT T-3’) and NTRTr/430 RV (5’-ACC GGC AAA CAC AAT TGT CA-3’) primer pair to obtain the 5’UTR sequence. Amplification products of 897 and 686 bp were obtained. Cloning and sequencing of the 686-bp amplicon revealed the *T. rangeli* Tre type I *NTR* 5’UTR region sequence, which consists of 257 nt (accession number KC797657.1) and shows 51% identity with the *T. cruzi* orthologous region of the 058PUJ isolate. Thus, type I *NTR* messenger is processed and expressed in *T. rangeli* epimastigotes as a mature mRNA.

Bioinformatics analysis revealed that the type I NTR from *T. rangeli* is a protein with a theoretical molecular mass of approximately 35 kDa. It is composed of an N-terminal region (amino acid residues 1-86) and a nitroreductase catalytic domain (amino acid residues 88-289). Because type I NTR enzymes were first described in bacteria and more recently in early protozoa, we compared N-terminal regions among several organisms (Fig. 1A, Supplementary data, Table I). The results indicated that in contrast to bacteria, protozoa possess a pre-sequence of the mature protein, which in the case of trypanosomatids contains a mitochondrial targeting signal (Fig. 1A). This pre-sequence likely appeared during protozoan evolution owing to the need to import the protein into the mitochondria for its biological function. Notably, although *Giardia*, *Trichomonas*, and *Entamoeba* do not possess mitochondria, they are equipped with mitosomes: a form of mitochondria lacking a genome and in which the protein import machinery is an incomplete vestigial derivative of mitochondrial components (Nyindodo-Ogari et al. 2014). This observation could explain the apparent lack of targeting signals in the type I NTR sequences from these organisms.

**Fig. 1:**
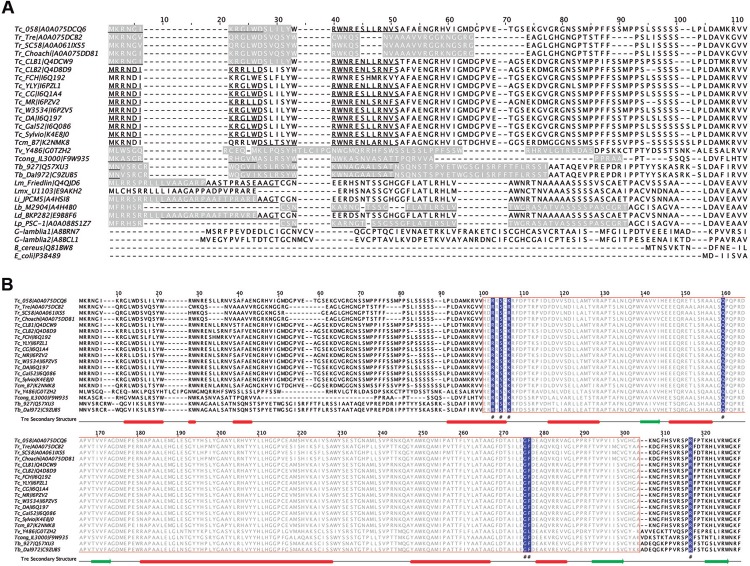
: multiple alignment-using Geneious (geneious.com) and Cobalt (ncbi.nlm.nih.gov/tools/cobalt/) - of the deduced type I nitroreductase (NTR) amino acid sequences from trypanosomatids, other protozoa, and bacteria (sequences shown in Supplementary data, Table I). (A) Alignment of the protein N-terminal region used for subcellular localisation prediction by 12 Web servers (Supplementary data, Table II). The MitoProt II- and iPSort-predicted mitochondrial targeting signal sequences are highlighted in grey and underlined, respectively. (B) Alignment of the complete proteins indicating domains according Interpro and Pfam (uniprot.org). The NTR domain is indicated by the red box and grey letters. The amino acid residues involved in the flavin mononucleotide (FMN) interaction are highlighted in blue (#). The regions implicated in the secondary structure were predicted using J pred in Jalview (Waterhouse et al. 2009) and are indicated as follows: helices are shown as red rectangles, and beta sheets are shown as green arrows.

As a preliminary approach to elucidating the mitochondrial location of the type I NTR protein from the Tre, Choachí, and SC58 strains, 12 servers for prediction using various algorithms were employed (Supplementary data, Table II). To analyse the obtained results, various numerical values were arbitrarily assigned: 1.0 to sequences with a high probability of being mitochondrial, 0.5 to those with medium probability, and 0 to those with zero probability. The results from the 12 servers were combined and are presented in Table. Although the experimentally determined mitochondrial *T. brucei* protein sequence exhibited a value of 9.5 (Wilkinson et al. 2008), those from cytoplasmic *T. rangeli* and *T. cruzi* calcineurin B, as expected, had values of 0. The type I NTR proteins from the *T. rangeli* Tre, SC58, and Choachí strains had values of 4, 5, and 7, respectively, indicating that only the Choachí sequence appears to be a mitochondrial protein. Indeed, analysis of the N-terminal amino acid composition of the *T. brucei* protein showed that this region is rich in positively charged amino acids but poor in negatively charged amino acids, a composition bias that is absent in the SC58 and Tre sequences (Supplementary data, Fig. 1). However, not all mitochondrial proteins have the typical N-terminal signal (Zhang et al. 2010), and thus we cannot rule out mitochondrial locations of the type I NTR proteins of these strains.

**TABLE:** : Prediction of subcellular localisation

Sequence	WolfPsort	Predotar	MitoProt II	PredSL	PSORT II	TargetP 1.0.1.0	iPSort	Euk-mPLoc	ESLpred	Cello v.2.5	SubLoc v.1.0.0	YLoc	Sum
Tc_058	1.0	0	1.0	0	0	1.0	1.0	0	1.0	0	1.0	0	**6.0**
Tr_Tre	1.0	0	1.0	0	0	0	0	0	0.5	1.0	0.5	0	**4.0**
Tr_SC58	1.0	0	1.0	0	0	1.0	0	0	0.5	1.0	0.5	0	**5.0**
Tr_Choachi	1.0	0.5	1.0	0	1.0	1.0	0	0	1.0	1.0	0.5	0	**7.0**
Tc_CLB1.0	1.0	0	1.0	0	0	0	1.0	0	1.0	1.0	1.0	0	**6.0**
Tc_CLB2	1.0	0.5	0.5	0	0	1.0	1.0	0.5	1.0	0	0.5	1.0	**7.0**
Tc_FCH	1.0	0	0.5	0	0	1.0	0	0	1.0	1.0	1.0	0.5	**6.0**
Tc_YLY	1.0	0	0.5	0	0	0	1.0	0	1.0	0	0.5	0.5	**4.5**
Tc_CG	1.0	0	0.5	0	0	0	1.0	0	1.0	0	0.5	0.5	**4.5**
Tc_MR	1.0	0.5	0.5	0	0	1.0	1.0	0	1.0	0	0.5	1.0	**6.5**
Tc_W3534	1.0	0	0.5	0	0	0	1.0	0	1.0	1.0	0.5	0.5	**5.5**
Tc_DA	1.0	0	0.5	0	0	0	1.0	0	1.0	1.0	0.5	0.5	**5.5**
Tc_Gal52	1.0	0	0.5	0	0	1.0	1.0	0	1.0	0	0.5	0.5	**5.5**
Tc_Sylvio	0	0	0.5	0	0	0	1.0	0	1.0	0	1.0	0	**3.5**
Tcm_B7	0	0	0.5	0	0	1.0	1.0	0	1.0	0	0.5	0	**4.0**
Tv_Y486	1.0	0	0.5	0	1.0	0	0	0	0.5	1.0	1.0	0	**5.0**
Tcg_IL3000	1.0	1.0	1.0	1.0	1.0	1.0	1.0	0	0.5	1.0	1.0	0.5	**10.0**
Tb_927	1.0	0.5	1.0	1.0	1.0	1.0	1.0	0	0.5	1.0	0.5	1.0	**9.5**
Tb_Dal972	1.0	0.5	1.0	1.0	1.0	1.0	1.0	0	0.5	1.0	0.5	1.0	**9.5**
Lm_Friedlin	1.0	1.0	1.0	1.0	1.0	1.0	1.0	1.0	0	0	0.5	1.0	**9.5**
Lmx_U1.01.003	1.0	0.5	0	0	0	1.0	0	0	0	0	1.0	0.5	**5.0**
Li_JPCM5	1.0	1.0	1.0	1.0	1.0	1.0	1.0	0	0	0	0.5	1.0	**7.5**
Lb_M2904	1.0	0	1.0	1.0	1.0	1.0	1.0	0	0	0	0.5	1.0	**7.5**
Ld_BPK282A1.0	1.0	1.0	1.0	1.0	1.0	1.0	1.0	0	0	0	0.5	1.0	**8.5**
Lp_PSC-1.0	1.0	0	1.0	1.0	1.0	1.0	1.0	1.0	0	0	0.5	0.5	**8.0**
Tc_CalB	0	0	0	0	0	0	0	0	na	0	0	0	**0**
Tr_CalB	0	0	0	0	0	0	0	0	na	0	0	0	**0**

Results are presented according to each program. An arbitrary value was assigned according to the prediction probability: 1.0 ≥ 90%; 50 ≤ 0.5 ≤ 90; 0 = not mitochondrial (in the case of MitoProt II, 0.5 corresponds to sequences without a signal sequence but predicted as mitochondrial). The last two rows shown two non-mitochondrial proteins, calcineurin B from *Trypanosoma cruzi* and *T. rangeli*. na: not available.

In contrast to the N-terminal region, alignment of all the *T. rangeli* type I NTR-deduced amino acid sequences (Tre, SC58, and Choachí strains) with those from other trypanosomatids showed that the type I NTR catalytic domain is highly conserved (Fig. 1B). Remarkably, *T. rangeli* type I NTRs from all the strains exhibited conservation of the NTR domain and critical residues involved in FMN interactions: R74, S76, R78, Q131, G247, and F248 (Fig. 2B). In addition, a structural homology model (PMDB ID: PM0080571, https://bioinformatics.cineca.it/PMDB/) was constructed from amino acid residues 65 to 298 (Fig. 2A). The N-terminal region between amino acid residues 32 and 68 is particularly rich in glycine (G) and proline (P), which can cause high structural entropy. The results of the structural assessment showed quality parameters within expected ranges for a good model (as shown in Supplementary data, Fig. 2). To establish structural similarity, the model was compared with experimental protein structures belonging to the same family (PDB ID: 3GFA, 2B67, and 1NOX) using PDBsum and PDBeFold. The NTR domain was structurally well conserved, with high similarity in the topology of secondary structural elements (Fig. 2B). Local docking analysis conducted by restricting binding to the binding site allowed us to observe the conserved amino acid residues involved in the FMN interaction. Blind docking analysis of the Tre-NTR model with its FMN cofactor showed the expected binding site as the location with higher-frequency ligand binding (Fig. 2C, the figure appears as a crowded site with all conformations superimposed). All these data indicate oxidoreductase activity for the predicted *T. rangeli* NTR protein.

**Fig. 2:**
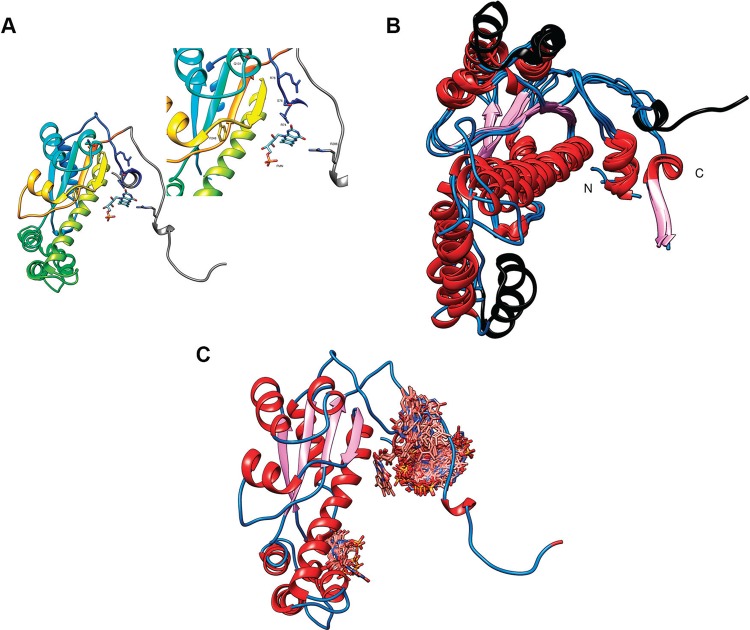
: *in silico* structural predictions for *Trypanosoma rangeli* type I nitroreductase (NTR) (PMDB ID: PM0080571). (A) A structural homology model was obtained by means of the I-Tasser server (zhanglab.ccmb.med.umich.edu/I-TASSER/), and its quality assessment was performed by several servers (swissmodel.expasy.org, prosa.services.came.sbg.ac.at/prosa.php, and ebi.ac.uk/thornton-srv/databases/cgi-bin/pdbsum/). Flavin mononucleotide (FMN) and the critical amino acid residues involved in binding are indicated as sticks. The residues are numbered according to the Tre sequence. (B) A comparison of the homology model of the *T. rangeli* type I NTR with similar proteins was conducted by superimposing it onto the following PDB IDs in PDBeFOLD (ebi.ac.uk/msd-srv/ssm/): 3GFA (Putative NTR from *Clostridium difficile*), 2B67 (NTR of *Streptococcus pneumoniae*), and 1NOX (NADH oxidase from *Thermus thermophilus*). Alpha helices are red, and beta sheets are purple. The black elements are not superimposed. The N- and C-terminal regions are indicated. (C) A homology model of the *T. rangeli* type I NTR was used to perform blind docking on the SwissDock server (swissdock.ch) using FMN as a ligand (Zinc entry 08551105) and was analysed using the Chimera package (Pettersen et al. 2004). FMN is observed in two sites; the site with the most frequently bound ligand (potential orientations are superimposed) corresponds to the reported binding site. The other site has a lower frequency of a ligand and may be an artefact because the structural model does not have the N-terminal residues (residues 1-64).

As a preliminary approach to assessment of the enzymatic activity of the *T. rangeli* type I NTR, epimastigote cultures of *T. rangeli* were exposed to benznidazole and nifurtimox, and parasite proliferation was analysed via an MTT assay using *T. cruzi* without a drug as a control. The effects of benznidazole and nifurtimox activation on parasite viability manifested themselves as a non-linear regression curve, which revealed a positive correlation between higher concentrations of the pro-drug and the dead parasite percentage (Fig. 3). The benznidazole IC50 for *T. rangeli* and *T. cruzi* was 21.5 and 32.4 µM, respectively, and the nifurtimox IC50 was 18.83 µM for *T. rangeli* and 14.75 µM for *T. cruzi*. Although both nitro-drugs were activated by the parasite, we did not demonstrate that NTR was responsible for this activation.

**Fig. 3:**
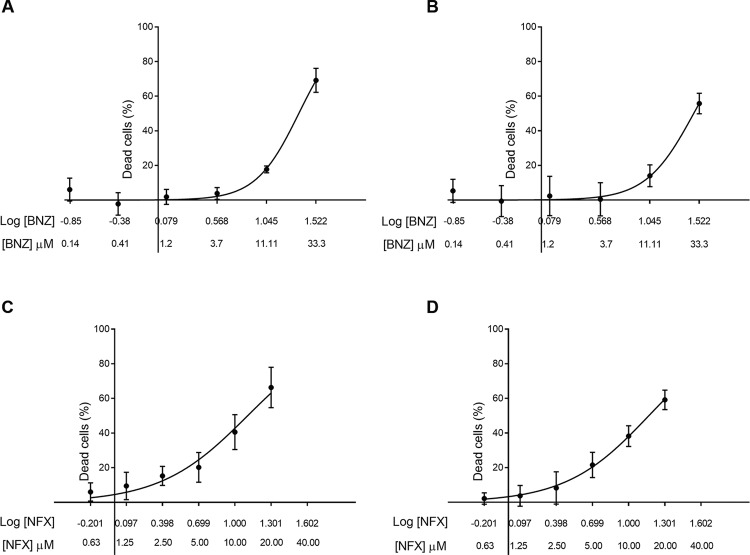
: benznidazole and nifurtimox activation. To examine the effect of benznidazole (N-benzyl-2-nitroimidazole-1-acetamide) or nifurtimox {4-thiomorpholinamine, 3-methyl-N-[(5-nitro-2-furanyl) methylene]-1,1-dioxide} on *Trypanosoma rangeli* (Tre strain) growth, the benznidazole or nifurtimox IC50 was determined in three independent experiments with three replicates each involving the MTT micromethod and *T. cruzi* (058PUJ isolate) as a control. The MTT assays showed that *T. rangeli* possesses the ability to activate nitro-drugs. (A-B) Representative non-linear regressions showing benznidazole pro-drug activation and subsequent parasite death for *T. rangeli* and *T. cruzi*, respectively. (C-D) Representative non-linear regressions showing pro-drug nifurtimox activation and subsequent parasite death for *T. rangeli* and *T. cruzi,* respectively.

In conclusion, *T. rangeli* appears to possess a functional type I NTR enzyme. The confirmation of its enzymatic activity and subcellular location could shed light on its biological role.
