# Supplementary material for: Identification of a type I nitroreductase gene in non-virulent Trypanosoma rangeli
Source: Mem Inst Oswaldo Cruz. 2017 Jul;112(7):504–9. doi: 10.1590/0074-02760160532 (PMC5452488; doi:10.1590/0074-02760160532)
Supplement: Supplementary file 1 [file 0074-0276-mioc-112-7-0504-suppl01.pdf]

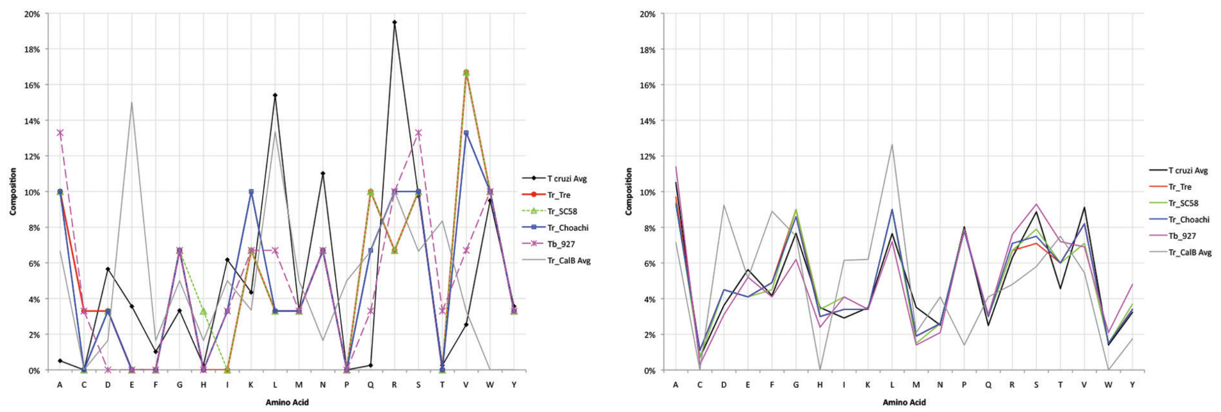

Fig. 1: amino acid composition of the N-terminal region (left panel) and remaining sequences (right panel) of type I NTR proteins. *Trypanosoma cruzi* values, presented as the average of all 11 sequences analysed, are black, and *T. rangeli* values are red (Tre), green (SC58), and blue (Choachí). *T. brucei*, presented as the average of two sequences, is pink, and the calcineurin B sequences from *T. cruzi* and *T. rangeli*, presented as averages, are grey.

TABLE I

Analysed type I NTR sequences with GenBank and Uniprot accession numbers

Label	Organism and classification	GenBank	Uniprot
Tc_058	*Trypanosoma cruzi* (DTU I)	AHI85558	A0A075DCQ6
Tr_Tre	*Trypanosoma rangeli* (KP1(-))	AHI85557	A0A075DCB2
Tr_SC58	*T. rangeli* (KP1(-))	ESL06666	A0A061IXS5
Tr_Choachi	*T. rangeli* (KP1(+))	AHI85556	A0A075DD81
Tc_CLB1	*T. cruzi* (DTU VI)	XP_810645	Q4D8D9
Tc_CLB2	*T. cruzi* (DTU VI)	XP_812226	Q4DCW9
Tc_FCH	*T. cruzi* (DTU II)	AFJ52880	I6Q192
Tc_YLY	*T. cruzi* (DTU I/II)	AFJ52881	I6PZL1
Tc_CG	*T. cruzi* (DTU II)	AFJ52882	I6Q1A4
Tc_MR	*T. cruzi* (DTU II)	AFJ52884	I6PZV2
Tc_W3534	*T. cruzi* (DTU I)	AFJ52889	I6PZV5
Tc_DA	*T. cruzi* (DTU I)	AFJ52890	I6Q197
Tc_Gal52	*T. cruzi* (DTU I)	AFJ52893	I6Q086
Tc_Sylvio	*T. cruzi* (DTU I)	EKG06918	K4E8J0
Tcm_B7	*T. cruzi*	EKF39034	K2NMK8
Tv_Y486	*Trypanosoma vivax*	CCC49376	G0TZH2
Tcg_IL3000	*Trypanosoma congolense*	CCD13725	F9W935
Tb_927	*Trypanosoma brucei*	XP_846343	Q57XU3
Tb_Dal972	*T. brucei gambiense* DAL972	XP_011775280	C9ZUB5
Lm_Friedlin	*Leishmania major*	XP_001687543	Q4QJD6
Lmx_U1103	*Leishmania mexicana*	XP_003871956	E9AKH2
Li_JPCM5	*Leishmania infantum* JPCM5	XP_001463029	A4HSI8
Lb_M2904	*Leishmania braziliensis*	XP_001561879	A4H4B0
Ld_BPK282A1	*Leishmania donovani*	XP_003858253	E9B8F6
Lp_PSC-1	*Leishmania panamensis*	XP_010703828	A0A088S1Z7
G-lamblia1	*Giardia lamblia*1	XP_001705312	A8BRN7
G-lamblia2	*G. lamblia*2	XP_001707931	A8BCL1
B. cereus	*Bacillus cereus*	AAP0997	Q81BW8
E. coli	*Escherichia coli*	NP_415110	P38489
Tc_CalB	*T. cruzi* calcineurin B	AEG15429	S4TLQ2
Tr_CalB	*T. cruzi* calcineurin B	AEG15431	S4TLX5

The model was evaluated using PDBSum, and the experimental structure was compared with those of other nitroreductases (NTR) proteins.

Plot statistics from Procheck based on Ramachandran plots		
Residues in most favored regions	202	81.1%
Residues in additional allowed regions	39	15.7%
Residues in generously allowed regions	4	1.6%
Residues in disallowed regions	4	1.6%

Topology diagram of secondary structure elements (SSE) from the PDBSum server

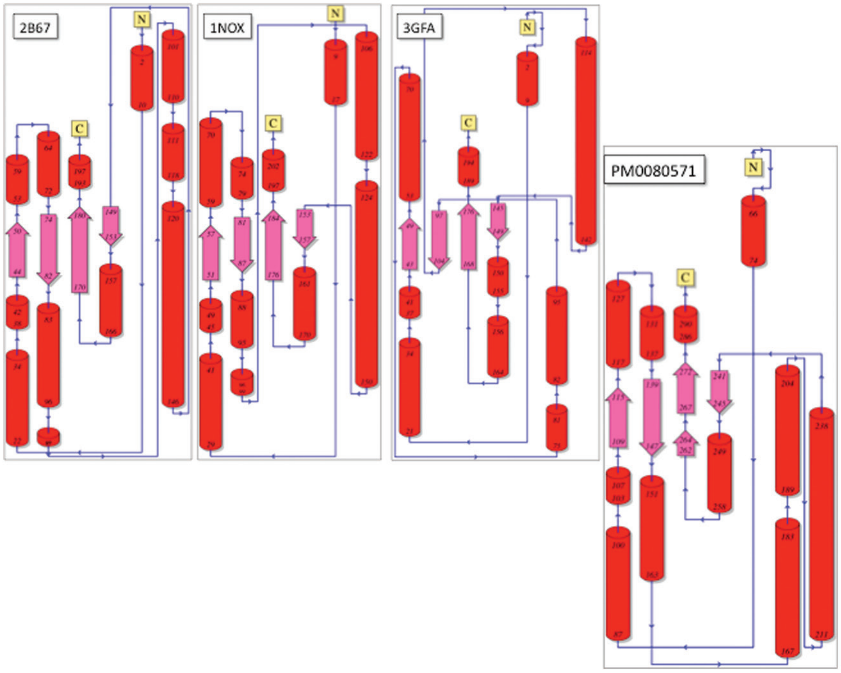

Helices are indicated as red cylinders, and beta sheets are indicated as pink arrows.

Summary of the PDBeFold results for comparison of the four structures

Number of aligned residues	147	Number of aligned SSEs	7
Overall RMSD	1.64	Overall Q-score	0.36

Secondary structure alignment (SSE)	
PM0080571 (Tre_NTR)	HHHShhshhhHs-HSh
1NOX_A	HHHShhs-hhHs-HSh
3GFA_A	HHHShh--hhHshHSh
2B67_D	HHHShhs-hhHs-HSh

RMSD \ Sequence identity (%)	Tre_NTR	1NOX_A	3GFA_A	2B67_D
Tre_NTR		34.0	16.3	25.9
1NOX_A	1.59		27.2	30.6
3GFA_A	1.86	1.89		26.5
2B67_D	1.54	1.39	1.46	

Fig. 2: structural model validation.

Supplementary data

TABLE II

Web servers used for subcellular localisation

Server	Web address
WolfPsort	http://www.genscript.com/wolf-psort.html
PREDOTAR	https://urgi.versailles.inra.fr/predotar/
MitoProt II	https://ihg.gsf.de/ihg/mitoprot.html
PredSL	http://aias.biol.uoa.gr/PredSL/
PSORT II	http://psort.hgc.jp
TargetP 1.1	http://www.cbs.dtu.dk/services/TargetP/
iPSort	http://ipsort.hgc.jp
Eukmploc	http://www.csbio.sjtu.edu.cn/bioinf/euk-multi-2/
ESLpred	http://www.imtech.res.in/raghava/eslpred2/submit.html
CELLO V.2.5 subCELlullar Localization predictor	http://cello.life.nctu.edu.tw
SubLoc v 1.0	http://www.bioinfo.tsinghua.edu.cn/SubLoc/
YLoc	http://abi.inf.uni-tuebingen.de/Services/YLoc/webloc.cgi
